# Maternal and neonatal complications after IVF/ICSI-fresh embryo transfer in low-prognosis women under the POSEIDON criteria: a retrospective cohort study

**DOI:** 10.1186/s12884-023-06176-2

**Published:** 2023-12-12

**Authors:** Shiguang Li, Huifang Tan, Huimin Fu, Juan Du, Peihao Liu, Yingying Qin

**Affiliations:** 1 Center for Reproductive Medicine, Cheeloo College of Medicine, Shandong University, Jinan, 250012 Shandong China; 2 Key Laboratory of Reproductive Endocrinology of Ministry of Education, Shandong University, Jinan, 250012 Shandong China; 3 Shandong Key Laboratory of Reproductive Medicine, Jinan, 250012 Shandong China; 4 Shandong Provincial Clinical Research Center for Reproductive Health, Jinan, 250012 Shandong China; 5 Shandong Technology Innovation Center for Reproductive Health, Jinan, 250012 Shandong China; 6 National Research Center for Assisted Reproductive Technology and Reproductive Genetics, Shandong University, Jinan, 250012 Shandong China; 7 Department of Obstetrics, Women’s Hospital, School of Medicine, Zhejiang University, Hangzhou, 310000 Zhejiang China

**Keywords:** POSEIDON criteria, Low prognosis, Maternal and neonatal complications, Advanced age, Poor ovarian reserve

## Abstract

**Background:**

Several studies on pregnancy complications of poor ovarian response (POR) patients did not draw a consistent conclusion. The POSEIDON criteria introduces the concept of “low prognosis” and divides POR patients into four groups based on age, AFC and AMH for individualized management. We analyzed low-prognosis population and patients with regular ovarian response, compared maternal and neonatal complications and discussed the relevant risk factors.

**Methods:**

A retrospective cohort study was conducted of females who achieved a singleton clinical pregnancy after IVF / ICSI—fresh embryo transfer in a single center from January 2014 to March 2019. Participants with low prognosis, as defined by the POSEIDON criteria, were enrolled in the study groups. The controls were defined as AFC ≥ five and number of retrieved oocytes > nine. Maternal and neonatal complications were compared among those groups.

**Results:**

There were 2554 cycles in POSEIDON group 1, 971 in POSEIDON group 2, 141 in POSEIDON group 3, 142 in POSEIDON group 4, and 3820 in Control. Univariate analysis roughly showed that Groups 2 and 4 had an increased tendency of pregnancy complications. Multi-variable generalized estimating equations (GEE) analysis showed that the risks of GDM, total pregnancy loss, and first-trimester pregnancy loss in Groups 2 and 4 were significantly higher than in Control. The risk of hypertensive disorders of pregnancy (HDP) in Groups 2 and 3 increased, and Group 4 had an increased tendency without statistical significance. After classification by age, GEE analysis showed no significant difference in risks of all complications among groups ≥ 35 years. In patients < 35 years, the risk of HDP in POSEIDON group 3 was significantly higher than in controls (< 35 years), and there was no significant increase in the risk of other complications.

**Conclusion:**

Compared to patients with regular ovarian response, low-prognosis population have increased tendency of maternal and neonatal complications. In low-prognosis patients, advanced age (≥ 35 years) might be the predominant risk factor for pregnancy complications. In those < 35 years, poor ovarian reserve could contribute to HDP.

**Supplementary Information:**

The online version contains supplementary material available at 10.1186/s12884-023-06176-2.

## Introduction

Poor ovarian response (POR) occurs when the ovary does not react adequately to gonadotropin (Gn) stimulation. As such, a larger dose of Gn is needed in POR patients undergoing assisted reproductive treatments. With POR, fewer developing follicles are generated, and blood estrogen peaks are lower. This often results in more frequent cycle cancellation, a smaller number of retrieved oocytes, and lower clinical pregnancy rates. POR has remained a complex and controversial clinical issue since first reported by Garcia [1]. The Bologna criteria, established by the European Society of Human Reproduction and Embryology (ESHRE) group, was the first criteria to standardize the definition of POR [1]. However, there are some clinical limitations of the Bologna criteria. In 2016, the Patient-Oriented Strategies Encompassing Individualized Oocyte Number (POSEIDON) criteria, which provides a more detailed stratification of poor responses that considers both age and ovarian reserve, were presented. This criteria also introduced the concept of "low prognosis" in POR patients [2]. According to the POSEIDON criteria, low-prognosis patients are divided into POSEIDON group 1, POSEIDON group 2, POSEIDON group 3, and POSEIDON group 4. Accordingly, clinicians have reported corresponding management strategies for clinical guidance of different groups under the POSEIDON criteria [3–5].

Pregnancy outcomes and complications of POR patients are widely studied. There are a series of studies on cumulative live birth rates (CLBRs) in POSEIDON populations [6–8]. Most of these investigations agreed that POSEIDON group 1 has the best pregnancy outcome, while POSEIDON group 4 has the most unsatisfactory outcome. The CLBR of younger patients (POSEIDON group 1 and POSEIDON group 3) was better than elder patients (POSEIDON group 2 and POSEIDON group 4). Research on pregnancy complications before and after the publication of the POSEIDON criteria reached different conclusions. Woldringh [9] found that the decrease in ovarian response to follicle-stimulating hormone (FSH) is related to the pathogenesis of pre-eclampsia in in-vitro fertilization (IVF) / intracytoplasmic sperm injection (ICSI) pregnancies. After excluding the influences of age and body mass index (BMI), his case–control study found that the total amount of Gn and FSH administered per day to the case group was higher than in the control group. They concluded that the decrease in ovarian response reflects the reduction of ovarian reserve, leading to a higher risk of pre-eclampsia. Jeroen van Disseldorp [10] classified IVF patients into a POR group (retrieved oocytes < 4) and regular ovarian response group (retrieved oocytes between 8 and 12). There was no significant difference between the two groups in terms of hypertensive disorders of pregnancy (HDP), pre-eclampsia, and newborn birth weight. Huriye [11] defined POR patients as those whose retrieved oocytes were less than six and matched cases and controls based on age. He found that the risks of gestational diabetes mellitus (GDM) and fetal disorders in POR patients did not increase. The limitation of this investigation was the small sample size. However, only one study has investigated pregnancy complications in low-prognosis patients. Raed K Abdullah and his colleagues [12] grouped patients according to the POSEIDON criteria to study CLBRs and pregnancy complications in a low-prognosis Chinese population. They found that the risks of HDP, GDM, placenta previa, abortion, and preterm delivery were not significantly different among four POSEIDON groups after adjustment for age and BMI.

Our aim in this study was to explore whether maternal and neonatal complications are associated with low prognosis by the POSEIDON criteria and identify the possible risk factors for these complications in four POSEIDON groups. To do so, we investigated the obstetric and perinatal outcomes per cycle after IVF / ICSI procedures, considering HDP, GDM, low birth weight (LBW), preterm birth (PTB), total pregnancy loss, first-trimester pregnancy loss, second-trimester pregnancy loss, induction due to malformation, placenta previa, and macrosomia as the primary outcomes.

## Materials and methods

### Participants

This study was a retrospective cohort of women who achieved a clinical singleton pregnancy after fresh embryo transfer in IVF / ICSI cycles at the Center for Reproductive Medicine, Shandong University, from January 2014 to March 2019. Low-prognosis patients as defined by POSEIDON criteria were enrolled in the study groups. Controls were defined as antral follicle count (AFC) ≥ five and number of retrieved oocytes > nine. We stratified BMI by the World Health Organization criteria [13] (< 18.5 kg/m^2^; ≥ 18.5 kg/m^2^, < 23 kg/m^2^; ≥ 23 kg/m^2^, < 27.5 kg/m^2^; ≥ 27.5 kg/m^2^). All data were obtained from the computerized medical record system in our center. Since advanced age exerts a significant adverse impact on maternal and neonatal complications, the participants were then stratified into two sets of groups, with the cutoff age of 35 years to further explore the relationship between low prognosis and various complications.

### Exclusion and inclusion criteria

Patients were excluded for any of the following reasons: polycystic ovary syndrome (PCOS, *n* = 2684); hypertension and preconceptional hypertension (*n* = 946); maternal or paternal chromosomal disorders (except polymorphism, *n* = 312); diabetes mellitus and preconceptional fasting glucose (PFG) ≥ 7.0 mmol/L (*n* = 135); preimplantation genetic testing (PGT, *n* = 123); gametes intrauterine transfer (GIUT, *n* = 79); donated oocytes (*n* = 140); heart, liver, kidney, and brain disorders (*n* = 82); multiple pregnancies (*n* = 4301); recurrent spontaneous abortion (*n* = 200); incomplete data (*n* = 625); lost to follow-up (*n* = 24); AFC ≥ 5 and antimullerian hormone (AMH) < 1.2 ng/ml (*n* = 1716); and AFC < 5 and AMH ≥ 1.2 ng/ml (*n* = 109).

Included cycles (*n* = 7628) were categorized based on POSEIDON criteria as follows.

1. Low prognosis patients:

POSEIDON group 1: Age < 35, AFC ≥ 5, AMH ≥ 1.2 ng/ml, number of retrieved oocytes ≤ 9, *n* = 2554.

POSEIDON group 2: Age ≥ 35, AFC ≥ 5, AMH ≥ 1.2 ng/ml, number of retrieved oocytes ≤ 9, *n* = 971.

POSEIDON group 3: Age < 35, AFC < 5, AMH < 1.2 ng/ml, *n* = 141.

POSEIDON group 4,: Age ≥ 35, AFC < 5, AMH < 1.2 ng/ml, *n* = 142.

(2) Non-low prognosis patients [7]:

Control: AFC ≥ 5, number of retrieved oocytes > 9, *n* = 3820. (Further categorized as Control 1 and Control 2. Control 1: Age < 35, *n* = 3102. Control 2: Age ≥ 35, *n* = 718.)

### Ovarian stimulation and IVF/ICSI

Every patient got a proper ovarian stimulation protocol; this included gonadotropin-releasing hormone (GnRH) agonist long protocols, GnRH agonist short protocols, GnRH antagonist protocol, and other protocols (natural cycle, modified natural cycle and mild ovarian stimulation). Other ovarian stimulation protocols included usage of recombinant FSH (Gonal-f, Merck Serono, Germany), GnRH antagonist (ganirelix, MSD Organon, the Netherlands), or urine-derived human menopausal gonadotropin (Menopur, Ferring, Switzerland), with individualized doses based on ultrasonography and endocrine status. When at least one follicle reached 18 mm in diameter, human chorionic gonadotrophin (HCG) at a dose of 4000—10,000 IU was administrated for final oocyte maturation triggering, followed by oocyte retrieval 24—36 h later. The type of fertilization was IVF or ICSI. According to morphologic scoring criteria [14–16], either two good-quality cleavage-stage embryos or one good-quality blastocyst-stage embryo was transferred. The luteal phase support includes daily oral dydrogesterone (Duphaston, Abbott Biologicals B.V., the Netherlands) and vaginal progesterone (Progesterone Soft Capsules, Cyndea Pharma, S.L., Spain) since oocyte retrieval.

### Assessment of primary outcomes

The primary outcomes included HDP, GDM, LBW, PTB, total pregnancy loss, first-trimester pregnancy loss, second-trimester pregnancy loss, induction due to malformation, placenta previa, and macrosomia. In our study, HDP included gestational hypertension and preeclampsia; chronic hypertension was excluded [17]. The diagnostic criteria for GDM included at least one of the following factors: fasting plasma glucose ≥ 7.0 mmol/L; 2 h plasma glucose ≥ 11.1 mmol/L following a 75 g oral glucose load; or random plasma glucose ≥ 11.1 mmol/L in the presence of diabetes symptoms [18]. PTB refers to a live birth before the gestational age of 37 weeks. Pregnancy loss was the spontaneous loss of pregnancy, classified as first-trimester pregnancy loss or second-trimester pregnancy loss [19]. Induction due to malformation was medically induced abortion caused by fetal malformation and chromosomal disorders, including 45 pregnancies with a severe fetal malformation (cheilopalatoschisis, rachischisis, severe congenital heart diseases etc.) and 18 pregnancies with abnormal fetal chromosomes (trisomy 21, trisomy 18, etc.) validated by prenatal diagnosis. Transvaginal ultrasound showing that the placenta lay directly over the internal os confirmed the diagnosis of placenta praevia [20]. Macrosomia was defined as a birth weight greater than 4000 g [21]. Vanishing twin syndrome refers to spontaneous reduction of a fetus while still in utero; the fetus either completely or partially disappears during pregnancy [22]. This study also considered those original multiple pregnancies that decreased to a singleton pregnancy as “vanishing twin syndrome.”

### Statistical analysis

Analyses in this study were conducted according to the type of variables (student’s t-test for dependent variables and median test for independent variables). Categorical variables were expressed as percentages and compared by Pearson’s Chi-square test. Pregnancy complications and perinatal outcomes were compared by their probabilities utilizing Pearson’s Chi-square test. Continuous variables comparison among these groups was analyzed using either the Analysis of variance (ANOVA) or the Median test. The odds ratio (OR) of pregnancy complications and the perinatal outcome was calculated as crude. Moreover, a generalized estimating equation (GEE) model using the control as reference was created and primarily adjusted to confounding factors that included BMI, cycles, type of fertilization, causes of infertility, type of infertility, ovarian stimulating protocol, HCG day estradiol (E_2_), HCG day progesterone (P), HCG day endometrium thickness (EM), and vanishing twin syndrome. All statistical analyses were done utilizing the computer software SPSS Version 26.0 (IBM, USA) at a 95% significance level.

## Results

### Pregnancy complications and obstetric outcomes in POSEIDON groups and controls

Table 1 shows the comparison of pregnancy complications and obstetric outcomes among the five groups. The GDM incidences in order from highest to lowest were 14.1% in POSEIDON group 4 (*n* = 20), 8.0% in POSEIDON group 2 (*n* = 78), 6.4% in POSEIDON group 3 (*n* = 9), 6.3% in POSEIDON group 1 (*n* = 160), and 5.2% in the control group (*n* = 199). POSEIDON group 4 had the highest incidences of total pregnancy loss, first-trimester pregnancy loss, and induction due to malformation, followed by POSEIDON group 2, control group, POSEIDON group 3, and POSEIDON group 1. For “vanishing twin syndrome” (including single live birth from triplets), ratios in all groups were different, and POSEIDON group 3 had the highest incidence (5.7%). There was no significant difference among groups in terms of LBW, PTB, second-trimester pregnancy loss, PP, or macrosomia.

**Table 1:** Maternal and neonatal complications

	POSEIDON Group 1 (*n* = 2554)	POSEIDON Group 2 (*n* = 971)	POSEIDON Group 3 (*n* = 141)	POSEIDON Group 4 (*n* = 142)	Control (*n* = 3820)	*P* value
**vanishing twin syndrome**	106(4.2)	32(3.3)^a^	8(5.7)	1(0.7)^abd^	187(4.9)	0.031
**GDM**	160(6.3)	78(8.0)^a^	9(6.4)	20(14.1)^abcd^	199(5.2)	< 0.001
**HDP**	58(2.3)	39(4.0)^ab^	7(5.0)	6(4.2)	92(2.4)	0.010
**LBW**	78(3.3)^a^	32(3.9)	6(4.7)	2(1.8)	151(4.3)	0.249
**PTB**	94(3.7)	37(3.8)	6(4.3)	6(4.2)	146(3.8)	0.993
**Pregnancy loss**						
Total pregnancy loss	159(6.2)^a^	126(13.0)^ab^	9(6.4)^c^	28(19.7)^abcd^	289(7.6)	< 0.001
1st trimester pregnancy loss	100(3.9)^a^	108(11.1)^ab^	6(4.3)^c^	26(18.3)^abcd^	211(5.5)	< 0.001
2nd trimester pregnancy loss	59(2.3)	18(1.9)	3(2.1)	2(1.4)	78(2.0)	0.910
**Induction due to malformation**	15(0.6)	16(1.6)^ab^	1(0.7)	4(2.8)^ab^	28(0.7)	0.005
**Placenta previa**	29(1.1)	14(1.4)	3(2.1)	3(2.1)	46(1.2)	0.473
**Macrosomia**	227(9.6)	97(11.7)	16(12.4)	14(12.6)	353(10.1)	0.348

Data are mean ± SD, median (interquartile), or n(%)

^a^*p* < 0.05, vs. Control

^b^*p* < 0.05, vs. POSEIDON group 1

^c^*p* < 0.05, vs. POSEIDON group 2

^d^*p* < 0.05, vs. POSEIDON group 3

We performed a multi-variable GEE analysis (Table 2) with variables that might act as confounding factors, which are described in Supplement Tables 1 and 2. The results showed that the risks of GDM, total pregnancy loss, and first-trimester pregnancy loss in POSEIDON group 2 and POSEIDON group 4 were significantly higher than in controls, consistent with trends of non-adjusted results. The risks of HDP in POSEIDON group 2 and POSEIDON group 3 were significantly higher, and POSEIDON group 4 had an increased tendency [OR 2.306, 95% confidence interval (CI) 0.82–6.484, *P* = 0.113] which did not reach statistical significance. Furthermore, the risk of LBW in POSEIDON group 1 was significantly lower than in controls (OR 0.723, 95% CI 0.529–0.988, *P* = 0.042), while there was no significance in other groups. There was no significant increase in the risks of other complications.

**Table 2:** Results of GEE analysis

	*P* value	Crude OR (95% CI)	*P* value	GEE Adjusted OR (95% CI)
**GDM**				
Control		1		1
POSEIDON Group 1	0.074	1.216(0.981, 1.507)	0.309	1.133(0.891, 1.442)
POSEIDON Group 2	0.001	1.589(1.211, 2.086)	0.041	1.398(1.014, 1.927)
POSEIDON Group 3	0.540	1.241(0.622, 2.474)	0.671	1.201(0.516, 2.794)
POSEIDON Group 4	< 0.001	2.983(1.820, 4.888)	0.005	2.690(1.353, 5.351)
**HDP**				
Control		1		1
POSEIDON Group 1	0.723	0.942(0.675, 1.313)	0.928	1.017(0.704, 1.469)
POSEIDON Group 2	0.007	1.696(1.158, 2.483)	0.010	1.781(1.148, 2.763)
POSEIDON Group 3	0.062	2.117(0.963, 4.653)	0.018	2.830(1.198, 6.685)
POSEIDON Group 4	0.177	1.788(0.769, 4.155)	0.113	2.306(0.820, 6.484)
**LBW**				
Control		1		1
POSEIDON Group 1	0.048	0.755(0.572, 0.998)	0.042	0.723(0.529, 0.988)
POSEIDON Group 2	0.561	0.891(0.604, 1.315)	0.970	1.008(0.659, 1.543)
POSEIDON Group 3	0.857	1.080(0.468, 2.490)	0.537	1.337(0.532, 3.356)
POSEIDON Group 4	0.210	0.406(0.099, 1.660)	0.717	0.769(0.186, 3.186)
**PTB**				
Control		1		1
POSEIDON Group 1	0.771	0.962(0.738, 1.252)	0.550	0.912(0.675, 1.232)
POSEIDON Group 2	0.987	0.997(0.690, 1.440)	0.569	1.126(0.748, 1.697)
POSEIDON Group 3	0.793	1.118(0.486, 2.576)	0.806	1.136(0.410, 3.147)
POSEIDON Group 4	0.806	1.110(0.482, 2.557)	0.502	1.420(0.510, 3.954)
**Total pregnancy loss**				
Control		1		1
POSEIDON Group 1	0.041	0.811(0.664, 0.991)	0.220	0.867(0.689, 1.090)
POSEIDON Group 2	< 0.001	1.822(1.459, 2.275)	< 0.001	1.887(1.449, 2.458)
POSEIDON Group 3	0.602	0.833(0.420, 1.654)	0.325	0.602(0.219, 1.654)
POSEIDON Group 4	< 0.001	3.001(1.951, 4.615)	< 0.001	3.610(2.044, 6.376)
**1**^**st**^** trimester pregnancy loss**				
Control		1		1
POSEIDON Group 1	0.004	0.697(0.546, 0.889)	0.028	0.733(0.555, 0.967)
POSEIDON Group 2	< 0.001	2.141(1.678, 2.731)	< 0.001	2.230(1.673, 2.971)
POSEIDON Group 3	0.517	0.760(0.332, 1.742)	0.411	0.614(0.192, 1.962)
POSEIDON Group 4	< 0.001	3.834(2.451, 5.997)	< 0.001	4.581(2.547, 8.242)
**2**^**nd**^** trimester pregnancy loss**				
Control		1		1
POSEIDON Group 1	0.470	1.134(0.806, 1.597)	0.258	1.256(0.846, 1.866)
POSEIDON Group 2	0.709	0.906(0.540, 1.520)	0.663	0.865(0.450, 1.661)
POSEIDON Group 3	0.944	1.043(0.325, 3.345)	0.605	0.592(0.081, 4.322)
POSEIDON Group 4	0.600	0.685(0.167, 2.817)	0.761	0.734(0.100, 5.373)
**Induction due to malformation**				
Control		1		1
POSEIDON Group 1	0.487	0.800(0.426, 1.501)	0.936	0.972(0.482, 1.958)
POSEIDON Group 2	0.009	2.269(1.223, 4.211)	0.095	1.963(0.890, 4.329)
POSEIDON Group 3	0.974	0.967(0.131, 7.160)	0.628	1.646(0.219, 12.397)
POSEIDON Group 4	0.012	3.925(1.358, 11.345)	0.489	2.041(0.270, 15.410)
**Placenta Previa**				
Control		1		1
POSEIDON Group 1	0.803	0.942(0.59, 1.504)	0.942	0.983(0.609, 1.586)
POSEIDON Group 2	0.553	1.200(0.657, 2.192)	0.556	1.206(0.646, 2.253)
POSEIDON Group 3	0.337	1.784(0.548, 5.805)	0.288	1.899(0.582, 6.199)
POSEIDON Group 4	0.343	1.771(0.544, 5.763)	0.283	1.913(0.586, 6.245)
**Macrosomia**				
Control		1		1
POSEIDON Group 1	0.527	0.945(0.793, 1.126)	0.272	0.896(0.737, 1.090)
POSEIDON Group 2	0.170	1.182(0.931, 1.501)	0.766	1.044(0.787, 1.384)
POSEIDON Group 3	0.398	1.259(0.738, 2.150)	1.000	1.000(0.496, 2.015)
POSEIDON Group 4	0.391	1.284(0.725, 2.273)	0.965	0.981(0.418, 2.302)

*OR* Odds ratio. OR was adjusted for group, female BMI, cycles, Type of fertilization, Causes of infertility, Type of infertility, Ovarian stimulating protocol, HCG day E2, HCG day P, HCG day EM, Vanishing twin syndrome. (For GDM, basal blood glucose plus. For HDP, basal blood pressure and GDM plus. For pregnancy loss, PTB and macrosomia, GDM plus. For LBW, PTB plus.)

### Pregnancy complications and obstetric outcomes in patients < 35 years old

Table 3 compares pregnancy complications and obstetric outcomes among patients < 35 years old, including POSEIDON group 1, POSEIDON group 3, and Control 1 group. The GDM incidences from highest to lowest were significantly different, 6.4% in POSEIDON group 3, 6.3% in POSEIDON group 1, and 4.7% in Control 1 group. POSEIDON group 3 had the highest incidence of HDP (5.0%), and Control 1 group had the lowest risk (2.0%). There was no significant difference among groups in terms of other pregnancy complications.

**Table 3:** Maternal and neonatal complications of participants < 35 years

	POSEIDON Group 1(*n* = 2554)	POSEIDON Group 3(*n* = 141)	Control 1 (*n* = 3102)	*P* value
**Vanishing twin syndrome**	106(4.2)	8(5.7)	151(4.9)	0.358
**GDM**	160(6.3)^a^	9(6.4)	145(4.7)	0.028
**HDP**	58(2.3)	7(5.0)^a^	61(2.0)	0.060
**LBW**	78(3.3)	6(4.7)	119(4.1)	0.234
**Preterm delivery**	94(3.7)	6(4.3)	122(3.9)	0.855
**Pregnancy loss**				
Total pregnancy loss	159(6.2)	9(6.4)	185(6.0)	0.910
First trimester pregnancy loss	100(3.9)	6(4.3)	124(4.0)	0.972
Second trimester pregnancy loss	59(2.3)	3(2.1)	61(2.0)	0.659
**Induction due to malformation**	15(0.6)	1(0.7)	20(0.6)	0.791
**Placenta previa**	29(1.1)	3(2.1)	36(1.2)	0.456
**Macrosomia**	227(9.6)	16(12.4)	288(10.0)	0.563

Data are mean ± SD, median (interquartile), or n (%)

^a^*p* < 0.05, vs. Control 1

^b^*p* < 0.05, vs. POSEIDON group 1

We performed GEE analysis (Table 4) according to Supplement Tables 3 and 4. Results showed that the risk of HDP in POSEIDON group 3 was significantly higher than that in Control 1 group (OR 3.599, 95% CI 1.499–8.642, *P* = 0.004). The risk of GDM and other outcomes of POSEIDON group 3 were not significantly increased compared to Control 1. In addition, there was no significant difference between POSEIDON group 1 and Control 1 group in all complications after adjustment.

**Table 4:** Results of GEE analysis of participants < 35 years

	*P* value	Crude OR (95% CI)	*P* value	GEE Adjusted OR (95% CI)
**GDM**				
Control 1		1		1
POSEIDON Group 1	0.009	1.3639(1.082, 1.717)	0.118	1.228(0.949, 1.588)
POSEIDON Group 3	0.353	1.39(0.694, 2.787)	0.544	1.301(0.556, 3.040)
**HDP**				
Control 1		1		1
POSEIDON Group 1	0.428	1.158(0.806, 1.666)	0.213	1.293(0.863, 1.938)
POSEIDON Group 3	0.019	2.604(1.169, 5.802)	0.004	3.599(1.499, 8.642)
**LBW**				
Control 1		1		1
POSEIDON Group 1	0.122	0.795(0.594, 1.063)	0.136	0.780(0.563, 1.081)
POSEIDON Group 3	0.766	1.136(0.491, 2.631)	0.438	1.442(0.572, 3.638)
**PTB**				
Control 1		1		1
POSEIDON Group 1	0.622	0.933(0.709, 1.228)	0.515	0.902(0.660, 1.231)
POSEIDON Group 3	0.848	1.086(0.47, 2.508)	0.824	1.123(0.404, 3.120)
**Total pregnancy loss**				
Control 1		1		1
POSEIDON Group 1	0.682	1.047(0.841, 1.303)	0.382	1.118(0.871, 1.435)
POSEIDON Group 3	0.837	1.075(0.538, 2.146)	0.625	0.776(0.281, 2.144)
**1**^**st**^** trimester pregnancay loss**				
Control 1		1		1
POSEIDON Group 1	0.875	0.979(0.748, 1.28)	0.865	1.027(0.756, 1.394)
POSEIDON Group 3	0.879	1.067(0.462, 2.466)	0.801	0.861(0.268, 2.769)
**2**^**nd**^** trimester pregnancay loss**				
Control 1		1		1
POSEIDON Group 1	0.373	1.179(0.821, 1.693)	0.219	1.299(0.856, 1.972)
POSEIDON Group 3	0.893	1.084(0.336, 3.497)	0.629	0.612(0.083, 4.490)
**Induction due to malformation**				
Control 1		1		1
POSEIDON Group 1	0.784	0.91(0.465, 1.782)	0.855	1.072(0.509, 2.258)
POSEIDON Group 3	0.926	1.101(0.147, 8.26)	0.566	1.816(0.237, 13.892)
**Placenta Previa**				
Control 1		1		1
POSEIDON Group 1	0.93	0.978(0.598, 1.6)	0.796	1.072(0.632, 1.819)
POSEIDON Group 3	0.31	1.851(0.563, 6.086)	0.415	1.825(0.430, 7.751)
**Macrosomia**				
Control 1		1		1
POSEIDON Group 1	0.663	0.96(0.799, 1.153)	0.362	0.910(0.743, 1.115)
POSEIDON Group 3	0.368	1.28(0.748, 2.191)	0.966	1.015(0.503, 2.050)

*OR* Odds ratio. OR was adjusted for group, cycles, Type of fertilization, Causes of infertility, Ovarian stimulating protocol, HCG day E2, HCG day P, HCG day EM, Vanishing twin syndrome. (For GDM, basal blood glucose plus. For HDP, basal blood pressure and GDM plus. For pregnancy loss, PTB and macrosomia, GDM plus. For LBW, PTB plus.)

### Pregnancy complications and obstetric outcomes in patients ≥ 35 years old

Table 5 compares pregnancy complications and obstetric outcomes among patients ≥ 35 years old, including POSEIDON group 2, POSEIDON group 4, and Control 2 group. The GDM incidences in order of highest to lowest were significantly different, 14.1% in POSEIDON group 4, 8.0% in POSEIDON group 2, and 7.5% in Control 2 group. POSEIDON group 4 had the highest incidences of total pregnancy loss (19.7%) and first-trimester pregnancy loss (18.3%); these incidences were significantly different from Control 2 group (14.5% and 12.1%). The ratios of “vanishing twin syndrome” were 3.3% in POSEIDON group 2, 0.7% in POSEIDON group 4, and 5.0% in Control 2 group. The risks of other complications were not significantly different.

**Table 5:** Maternal and neonatal complications of participants ≥ 35 years

	POSEIDON Group 2(*n* = 971)	POSEIDON Group 4(*n* = 142)	Control 2 (*n* = 718)	*P* value
**vanishing twin syndrome**	32(3.3)	1(0.7)^cd^	36(5.0)	0.025
**GDM**	78(8.0)	20(14.1)^cd^	54(7.5)	0.032
**HDP**	39(4.0)	6(4.2)	31(4.3)	0.953
**LBW**	32(3.9)	2(1.8)	32(5.3)	0.167
**Preterm delivery**	37(3.8)	6(4.2)	24(3.3)	0.820
**Pregnancy loss**				
Total pregnancy loss	126(13.0)	28(19.7)^d^	104(14.5)	0.091
First trimester pregnancy loss	108(11.1)	26(18.3)^cd^	87(12.1)	0.049
Second trimester pregnancy loss	18(1.9)	2(1.4)	17(2.4)	0.758
**Induction due to malformation**	16(1.6)	4(2.8)	8(1.1)	0.228
**Placenta previa**	14(1.4)	3(2.1)	10(1.4)	0.716
**Fetal macrosomia**	97(11.7)	14(12.6)	65(10.8)	0.793

Data are mean ± SD, median (interquartile), or n (%). cp < 0.05, vs. Control 2; dp < 0.05, vs. POSEIDON group 2

We performed GEE analysis (Table 6) according to Supplement Tables 5 and 6. Results showed that, after adjustment, there was no significant increase in risks of all complications in POSEIDON group 2 and POSEIDON group 4.

**Table 6:** Results of GEE analysis of participants ≥ 35 years

	*P* value	Crude OR (95% CI)	*P* value	GEE Adjusted OR (95% CI)
**GDM**				
Control 2		1		1
POSEIDON Group 2	0.698	1.074(0.748, 1.541)	0.890	1.031(0.669, 1.588)
POSEIDON Group 4	0.012	2.016(1.165, 3.487)	0.072	1.984(0.941, 4.184)
**HDP**				
Control 2		1		1
POSEIDON Group 2	0.759	0.927(0.573, 1.501)	0.623	0.872(0.504, 1.507)
POSEIDON Group 4	0.961	0.978(0.400, 2.389)	0.826	1.129(0.382, 3.337)
**LBW**				
Control 2		1		1
POSEIDON Group 2	0.194	0.717(0.434, 1.184)	0.245	0.722(0.418, 1.249)
POSEIDON Group 4	0.129	0.327(0.077, 1.384)	0.425	0.551(0.128, 2.379)
**PTB**				
Control 2		1		1
POSEIDON Group 2	0.611	1.146(0.679, 1.933)	0.553	1.194(0.665, 2.145)
POSEIDON Group 4	0.601	1.276(0.512, 3.180)	0.468	1.506(0.498, 4.551)
**Total pregnancy loss**				
Control 2		1		1
POSEIDON Group 2	0.372	0.880(0.666, 1.164)	0.399	0.867(0.622, 1.209)
POSEIDON Group 4	0.115	1.450(0.913, 2.303)	0.101	1.658(0.906, 3.031)
**1**^**st**^** trimester pregnancy loss**				
Control 2		1		1
POSEIDON Group 2	0.527	0.908(0.672, 1.226)	0.539	0.895(0.628, 1.276)
POSEIDON Group 4	0.048	1.626(1.005, 2.629)	0.055	1.838(0.986, 3.428)
**2**^**nd**^** trimester pregnancy loss**				
Control 2		1		1
POSEIDON Group 2	0.465	0.779(0.399, 1.522)	0.498	0.747(0.321, 1.737)
POSEIDON Group 4	0.482	0.589(0.135, 2.578)	0.665	0.634(0.081, 4.981)
**Induction due to malformation**				
Control 2		1		1
POSEIDON Group 2	0.363	1.487(0.633, 3.494)	0.587	1.356(0.452, 4.071)
POSEIDON Group 4	0.127	2.572(0.764, 8.661)	0.755	1.411(0.163, 12.24)
**Placenta Previa**				
Control 2		1		1
POSEIDON Group 2	0.933	1.036(0.457, 2.345)	0.668	1.214(0.500, 2.946)
POSEIDON Group 4	0.524	1.528(0.415, 5.623)	0.338	1.926(0.504, 7.353)
**Macrosomia**				
Control 2		1		1
POSEIDON Group 2	0.583	1.098(0.787, 1.532)	0.861	0.966(0.654, 1.426)
POSEIDON Group 4	0.576	1.192(0.644, 2.209)	0.832	0.908(0.371, 2.220)

*OR* Odds ratio. OR was adjusted for group, cycles, Ovarian stimulating protocol, HCG day E2, HCG day P, HCG day EM, Vanishing twin syndrome. (For GDM, basal blood glucose plus. For HDP, basal blood pressure and GDM plus. For pregnancy loss, PTB and macrosomia, GDM plus. For LBW, PTB plus.)

## Discussion

This study explored the risks of maternal and neonatal complications, including HDP, GDM, pregnancy loss, induction due to malformation, PTB, LBW, macrosomia, and placenta previa, in a low-prognosis population using POSEIDON criteria after IVF / ICSI treatment. POSEIDON groups 2 and 3 had increased risks of HDP, and POSEIDON group 4 had an elevated tendency toward HDP. POSEIDON groups 2 and 4 had significantly higher risks of GDM, total pregnancy loss, and first-trimester pregnancy loss.

The incidence of HDP in low-prognosis patients has not been widely reported previously. Only Raed showed that the incidences of HDP in POSEIDON groups varied from 2.3% to 5.8% [12]; the incidence in our study was 2.9% (110/3808) in low-prognosis patients. Advanced age is an independent risk factor for HDP [23, 24]. This could contribute to the higher incidences in POSEIDON groups 2 and 4.

Ovarian reserve might be associated with HDP. Previously, Han S et al. demonstrated that females with diminished ovarian reserve (DOR) had an increased incidence of HDP [25]. According to POSEIDON criteria, ovarian features of low-prognosis patients include impaired ovarian response based on sufficient ovarian reserve (POSEIDON groups 1 and 2) and poor ovarian reserve (POSEIDON groups 3 and 4). Poor ovarian reserve in POSEIDON criteria refers to AFC < 5 and AMH < 1.2 ng/mL. Compared to controls, the ovarian reserve of POSEIDON group 3 was diminished, and the risk of HDP was significantly increased. Some studies have proposed an underlying mechanism for this. Poor ovarian reserve and decreased ovarian reserve are clinical signs of ovarian aging. First, luteal phase dysfunction in ovarian aging is related to maternal vascular health. Luteal phase progesterone and estradiol levels and metabolite production are decreased in ovarian aging [26]. According to another study, the risk of HDP in hormone replacement therapy cycles without the corpus luteum is increased. Luteal phase dysfunction is supposedly associated with vascular problems and HDP [27]. Besides, a prospective longitudinal cohort study by Cavoretto [28] reveled that the risk of pre-eclampsia in IVF/ICSI singleton pregnancies with oocyte donation was significantly higher than in natural conception (6.6% and 0.6%, *P* = 0.003). The mean uterine artery pulsatility indexes at three trimesters were significantly lower than in natural conception. The relationship between HDP and luteum dysfunction could be partially attributed to relaxin, a vasodilator produced by the corpus luteum during pregnancy. Relaxin is a key factor in uterine arteries' compositional and geometric remodeling; it increases the level of inducible nitric oxide to achieve vasodilation. Consequently, low-prognosis patients with poor ovarian reserve could be complicated with ovarian aging and luteal phase dysfunction, causing a low level of relaxin in early pregnancy and increasing the risk of maternal vascular problems. However, the clinical application of relaxin is limited, and such insufficiency of relaxin couldn’t be supplemented by general luteal phase support.

Second, oxidative stress might lead to a higher risk of HDP in low-prognosis patients with poor ovarian reserve. Reactive oxygen species (ROS) are active compounds comprising oxygen; they include superoxide anions, hydrogen peroxide, and hydroxyl radicals. ROS are endogenously generated from cellular oxygen metabolism and overproduction of ROS causes oxidative stress and cellular damage [29]. Qualitative deterioration of ovarian follicles and oocytes in low-prognosis patients might be caused by such oxidative damage. In addition, oxidative stress could influence arterial remodeling and functional changes. Reduced vasodilation and increased vasoconstriction, that is, endothelial dysfunction, plays an important role in the pathogenesis of hypertension [30]. Excessive oxidative stress has been shown to be relevant to hypertension and vascular pathologies in both human and animal models [31].

GDM is the most common pregnancy complication and results in other complications such as PTB and macrosomia. The higher risks of GDM in POSEIDON groups 2 and 4 might be related to advanced age. A former large-sample study has demonstrated that advanced age raises the risk of GDM [32]. Such an age-related effect could be associated with deterioration of glucose-insulin regulation and endocrine function in elder women.

Many studies have elucidated that advanced maternal age is an independent risk factor for pregnancy loss in women older than 35 years old and adversely influences oocyte quality and chromosomal segregation errors (aneuploidy) [33, 34]. Age-associated oocyte aneuploidy likely leads to fetal chromosomal abnormalities, which are the main cause of first-trimester pregnancy loss [35]. That might be the reason for the higher risks of total pregnancy loss and first-trimester pregnancy loss in POSEIDON groups 2 and 4.

Our study can provide fertility doctors and obstetricians with new insight on gestational management of low-prognosis patients. HDP comprises maternal and neonatal morbidity and mortality [36]. It is essential to investigate the risk factors of HDP and take measures to prevent it. Since poor ovarian reserve might be a risk factor for HDP, evaluation of ovarian reserve and measurements of blood pressure could be more closely surveilled in a patient if she belonged to POSEIDON group 3. First, more frequent measurements of blood pressure at home could be performed. Weight gain during pregnancy should be under control, because obesity can increase the risk of preeclampsia. If necessary, administration of low-dose aspirin might be considered for the prevention of preeclampsia.

Recommendations for patients in POSEIDON groups 2 and 4 might include routine monitoring of blood glucose, a low carbohydrate diet, and regular physical activity. If necessary, oral hypoglycaemic agents and even insulin could be administrated to achieve normoglycemia. Low-prognosis females over 35 years old also have increased risk of pregnancy loss, especially within the first trimester. They might need intensive prenatal visits, including ultrasound examination and invasive prenatal diagnostic testing. Psychological treatment should be considered after excluding other disorders related with pregnancy loss such as antiphospholipid syndrome and luteal phase defect. Progesterone could be utilized to stabilize pregnancy if necessary.

Our study is the first to compare the maternal and fetal complications of low-prognosis patients and regular response patients (controls). We found that POSEIDON groups 2 and 4 had worse obstetrical prognosis among the four groups, consistent with the only previous report [12], in which there were no controls and the sample size was smaller than ours. We also found that poor ovarian reserve might increase HDP risk in a low-prognosis population. Compared to other studies, ours had different conclusions about the relationship between ovarian reserve and HDP risk; this could be attributed to different populations and inclusion criteria [36, 37]. Moreover, this study excluded cases whose AFC and AMH did not satisfy the POSEIDON criteria to weaken heterogeneity. The major limitation was the retrospective design. Going forward, a prospective study with a larger-size sample would be superior.

## Conclusion

In conclusion, POSEIDON groups 2 and 3 had increased risks of HDP, and POSEIDON group 4 had an elevated tendency toward HDP. POSEIDON groups 2 and 4 had significantly higher risks of GDM, total pregnancy loss, and first-trimester pregnancy loss. Age might the predominant risk factor for pregnancy complications (HDP, GDM, total pregnancy loss, and first-trimester pregnancy loss) in low-prognosis patients. Poor ovarian reserve might be associated with an increased risk of HDP in low-prognosis patients.

### Supplementary Information

**Additional file 1.**

**Additional file 2.**

**Additional file 3.**

**Additional file 4.**

**Additional file 5.**

**Additional file 6.**

## Data Availability

The datasets used during the current study are available from the corresponding authors on reasonable request.

## Abbreviations

AFC: Antral follicle counting
AMH: Anti-Müllerian hormone
ANOVA: One-way analysis variance
BMI: Body mass index
CI: Confidence interval
CLBR: Cumulative live birth rate
DOR: Diminished ovarian reserve
EM: Endometrium
ESHRE: European Society of Human Reproduction and Embryology
E_2_: Estradiol
FSH: Follicle-stimulating hormone
GDM: Gestational diabetes
GEE: Generalized estimating equations
GIUT: Gametes intrauterine transfer
Gn: Gonadotropin
HCG: Human chorionic gonadotropin
HDP: Hypertensive disease of pregnancy
ICSI: Intracytoplasmic sperm injection
IVF: In vitro fertilization
LBW: Low birth weight
LH: Luteinizing hormone
P: Progesterone
POR: Poor ovarian response
POSEIDON: Patient-oriented strategies encompassing individualized oocyte number
PTB: Preterm birth
ROS: Reactive oxygen species
TO: Testosterone
TSH: Thyroid-stimulating hormone
VTS: Vanishing twin syndrome
