# Supplementary material for: Maternal and neonatal complications after IVF/ICSI-fresh embryo transfer in low-prognosis women under the POSEIDON criteria: a retrospective cohort study
Source: BMC Pregnancy Childbirth. 2023 Dec 12;23:855. doi: 10.1186/s12884-023-06176-2 (PMC10714626; doi:10.1186/s12884-023-06176-2)
Supplement: Supplementary file 1 — Additional file 1. [file 12884_2023_6176_MOESM1_ESM.docx]

**Supplement table 1** Baseline characteristics of participants in different groups

|  | | POSEIDON Group 1  (n=2554) | | POSEIDON Group 2  (n=971) | POSEIDON Group 3  (n=141) | POSEIDON Group 4  (n=142) | Control  (n=3820) | P value |
| --- | --- | --- | --- | --- | --- | --- | --- | --- |
| **Age (years)** | | 29.6±2.9^a^ | | 37.4±2.3^ab^ | 30.4±2.8^bc^ | 38.3±2.5^abd^ | 30.6±4.2 | <0.001 |
| **AFC** | | 12(9, 15)^a^ | | 10(8, 14)^ab^ | 3(3, 4)^ab^ | 3(2, 4)^ab^ | 14(11, 17) | <0.001 |
| **AMH (ng/mL)** | | 2.8(1.9, 4.3)^a^ | | 2.3(1.7, 3.5)^ab^ | 0.4(0.3, 0.7)^ab^ | 0.4(0.2, 0.8)^ab^ | 3.6(2.4, 5.3) | <0.001 |
| **FSH (IU/L)** | | 6.9(6.0, 8.1)^a^ | | 7.1(6.0, 8.2)^ab^ | 9.3(7.3, 12.8)^abc^ | 8.7(6.9, 11.5)^ab^ | 6.4(5.5, 7.3) | <0.001 |
| **LH (IU/L)** | | 4.6(3.5, 6.0) | | 4.4(3.4, 5.7)^ab^ | 4.0(3.0, 5.9)^ab^ | 4.5(3.2, 6.1） | 4.7(3.5, 6.1) | <0.001 |
| **E2 (pg/mL)** | | 34.0(26.0, 45.7)^a^ | | 35.0(27.1, 47.8)^a^ | 37.5(24.6, 69.6)^ab^ | 42.3(26.1, 72.3)^ab^ | 32.7(24.8, 43.1) | <0.001 |
| **TO (ng/dL)** | | 22.5(16.1, 30.6) | | 20.0(14.1, 26.2)^ab^ | 20.5(12.8, 26.7)^ab^ | 18.3(12.5, 25.8)^ab^ | 22.1(15.9, 29.9) | <0.001 |
| **TSH (μIU/mL)** | | 2.2(1.6, 3.0) | | 2.1(1.4, 2.8)^a^ | 2.4(1.5, 3.1) | 2.2(1.6, 3.0) | 2.2(1.6, 3.0) | 0.014 |
| **Basal systolic blood pressure (mmHg)** | | | |  |  |  |  |  |
|  | | 116.7±11.8 | | 117.2±12.0 | 116.4±11.9 | 115.4±13.0 | 117.0±11.5 | 0.438 |
| **Basal diatolic blood pressure (mmHg)** | | | |  |  |  |  |  |
|  | 69.6±8.6^a^ | | 70.7±9.2^b^ | | 69.2±8.9 | 69.3±9.1 | 70.1±8.6 | 0.008 |
| **Basal blood glucose (mmol/L)** | | | |  |  |  |  |  |
|  | 5.20±0.46 | | 5.26±0.51^ab^ | | 5.24±0.48 | 5.28±0.46 | 5.20±0.45 | 0.016 |
| **BMI (kg/m^2^)** | |  | |  |  |  |  | <0.001 |
| BMI < 18.5 | | 168(6.6)^a^ | | 19(2.0)^ab^ | 8(5.7)^c^ | 2(1.4)^abc^ | 194(5.1) | <0.001 |
| 18.5 ≤ BMI < 23 | | 1202(47.1) | | 383(39.5)^ab^ | 65(46.1) | 56(39.4) | 1823(47.7) | <0.001 |
| 23 ≤ BMI < 27.5 | | 884(34.6) | | 431(44.4)^ab^ | 47(33.3)^c^ | 56(39.4)^c^ | 1376(36.0) | <0.001 |
| BMI ≥ 27.5 | | 300(11.7) | | 138(14.2)^a^ | 21(14.9) | 28(19.7)^ab^ | 427(11.2) | 0.003 |
| **Type of infertility** | | | |  |  |  |  |  |
| Primary | | 1427(55.9)^a^ | | 220(22.7)^ab^ | 76(53.9) | 34(23.9)^abcd^ | 1917(50.2) | <0.001 |
| Secondary | | 1127(44.1) | | 751(77.3) | 65(46.1) | 108(76.1) | 1903(49.8) |  |
| **Causes of infertility** | | | |  |  |  |  | <0.001 |
| Tubal factors | | 1776(69.5)^a^ | | 688(70.9)^a^ | 99(70.2) | 93(65.5) | 2546(66.6) | 0.037 |
| Male factors | | 443(17.3)^a^ | | 120(12.4)^ab^ | 15(10.6)^a^ | 13(9.2)^ab^ | 774(20.3) | <0.001 |
| Combined factors | | 32(1.2) | | 14(1.4) | 0(0.0) | 4(2.8) | 63(1.6) | 0.229 |
| Others | | 303(11.9) | | 149(15.3)^ab^ | 27(19.1)^ab^ | 32(22.5)^ab^ | 437(11.4) | <0.001 |
| **Type of fertilization** | | | |  |  |  |  |  |
| IVF | | 1830(71.7)^a^ | | 707(72.8)^a^ | 107(75.9)^a^ | 105(73.9) | 2547(66.7) | <0.001 |
| ICSI | | 724(28.3) | | 264(27.2) | 34(24.1) | 37(26.1) | 1273(33.3) |  |

Data are mean ± SD, median (interquartile), or n (%). ^a^p<0.05, vs. Control; ^b^p<0.05, vs. POSEIDON group 1; ^c^p<0.05, vs. POSEIDON group 2; ^d^p<0.05, vs. POSEIDON group 3.
