# Supplementary material for: Maternal and neonatal complications after IVF/ICSI-fresh embryo transfer in low-prognosis women under the POSEIDON criteria: a retrospective cohort study
Source: BMC Pregnancy Childbirth. 2023 Dec 12;23:855. doi: 10.1186/s12884-023-06176-2 (PMC10714626; doi:10.1186/s12884-023-06176-2)
Supplement: Supplementary file 2 — Additional file 2. [file 12884_2023_6176_MOESM2_ESM.docx]

**Supplement table 2** IVF/ICSI-technique parameters

|  | POSEIDON Group 1  (n=2554) | POSEIDON Group 2  (n=971) | POSEIDON Group 3  (n=141) | POSEIDON Group 4  (n=142) | Control  (n=3820) | P value |
| --- | --- | --- | --- | --- | --- | --- |
| **Ovarian stimulating protocol, n (%)** | | |  |  |  | <0.001 |
| Long GnRH agonist | 1434(56.1)^a^ | 445(45.8)^ab^ | 17(12.1)^abc^ | 22(15.5)^abc^ | 2710(70.9) | <0.001 |
| Short GnRH agonist | 594(23.3)^a^ | 406(41.8)^ab^ | 81(57.4)^abc^ | 69(48.6)^abc^ | 497(13.0) | <0.001 |
| GnRH antagonist | 472(18.5)^a^ | 102(10.5)^ab^ | 14(9.9)^b^ | 14(9.9)^b^ | 557(14.6) | <0.001 |
| Others | 54(2.1)^a^ | 18(1.9) | 29(20.6)^abc^ | 37(26.1)^abc^ | 56(1.5) | <0.001 |
| **HCG day E_2_ (pg/mL)** | 2140(1632, 2818)^a^ | 2190(1680, 2864)^a^ | 1355(648, 1859)^abc^ | 1062(492, 1642)^abd^ | 3273(2649, 4322) | <0.001 |
| **HCG day P (ng/mL)** | 0.7(0.5, 0.9)^a^ | 0.7(0.5, 0.9)^a^ | 0.6(0.4, 0.9)^abc^ | 0.6(0.3, 0.8)^ab^ | 0.8(0.6, 1.1) | <0.001 |
| **HCG day endometrial thickness (cm)** | 1.11±0.19^a^ | 1.08±0.20^ab^ | 1.09±0.23 | 1.01±0.18^abd^ | 1.13±0.25 | <0.001 |
| **Number of retrieved oocytes** | 7(5, 8)^a^ | 7(5, 8)^ab^ | 3(2, 5)^abc^ | 3(2, 5)^ab^ | 13(11, 15) | <0.001 |

Data are mean ± SD, median (interquartile), or n (%). ^a^p<0.05, vs. Control; ^b^p<0.05, vs. POSEIDON group 1; ^c^p<0.05, vs. POSEIDON group 2; ^d^p<0.05, vs. POSEIDON group 3.
