# Supplementary material for: Maternal and neonatal complications after IVF/ICSI-fresh embryo transfer in low-prognosis women under the POSEIDON criteria: a retrospective cohort study
Source: BMC Pregnancy Childbirth. 2023 Dec 12;23:855. doi: 10.1186/s12884-023-06176-2 (PMC10714626; doi:10.1186/s12884-023-06176-2)
Supplement: Supplementary file 3 — Additional file 3. [file 12884_2023_6176_MOESM3_ESM.docx]

**Supplement table 3** Baseline characteristics of participants < 35 years

|  | | | POSEIDON Group 1 (n=2554) | POSEIDON Group 3 (n=141) | Control 1 (n=3102) | P value |
| --- | --- | --- | --- | --- | --- | --- |
| **Age (years)** | | | 29.6±2.9^a^ | 30.4±2.8^ab^ | 29.1±2.9 | <0.001 |
| **AFC** | | | 12(9, 15)^a^ | 3(3, 4)^ab^ | 14(11, 17) | <0.001 |
| **AMH (ng/mL)** | | | 2.8(1.9, 4.3)^a^ | 0.4(0.3, 0.7)^ab^ | 3.6(2.4, 5.4) | <0.001 |
| **FSH (IU/L)** | | | 6.9(6.0, 8.1)^a^ | 9.3(7.3, 12.8)^ab^ | 6.3(5.5, 7.3) | <0.001 |
| **LH (IU/L)** | | | 4.6(3.5, 6.0)^a^ | 4.0(3.0, 5.9)^ab^ | 4.7(3.6, 6.2) | 0.001 |
| **E2 (pg/mL)** | | | 34.0(26.0, 45.7)^a^ | 37.5(24.6, 69.6)^ab^ | 32.8(25.0, 43.0) | <0.001 |
| **TO (ng/dL)** | | | 22.5(16.1, 30.6) | 20.5(12.8, 26.7)^ab^ | 22.8(16.4, 30.4) | 0.013 |
| **TSH (μIU/mL)** | | | 2.2(1.6, 3.0) | 2.4(1.5, 3.1) | 2.2(1.6, 3.0) | 0.542 |
| **Basal systolic blood pressure (mmHg)** | | | |  |  |  |
|  |  | | 116.7±11.8 | 116.4±11.9 | 116.8±11.5 | 0.884 |
| **Basal diatolic blood pressure (mmHg)** | | | |  |  |  |
|  |  | 69.6±8.6 | | 69.2±8.9 | 69.8±8.6 | 0.395 |
| **Basal blood glucose (mmol/L)** | | | |  |  |  |
|  |  | 5.20±0.46 | | 5.24±0.48 | 5.18±0.45 | 0.070 |
| **BMI (kg/m^2^)** | | |  |  |  | 0.214 |
| BMI<18.5 | | | 168(6.6) | 8(5.7) | 188(6.1) | 0.695 |
| 18.5≤BMI<23 | | | 1202(47.1) | 65(46.1) | 1535(49.5) | 0.167 |
| 23≤BMI<27.5 | | | 884(34.6) | 47(33.3) | 1067(34.4) | 0.946 |
| BMI≥27.5 | | | 300(11.7)^a^ | 21(14.9) | 312(10.1) | 0.040 |
| **Type of infertility** | | |  |  |  |  |
| Primary | | | 1427(55.9) | 76(53.9) | 1774(57.2) | 0.498 |
| Secondary | | | 1127(44.1) | 65(46.1) | 1328(42.8) |  |
| **Causes of infertility** | | |  |  |  | <0.001 |
| Tubal factors | | | 1776(69.5)^a^ | 99(70.2) | 2029(65.4) | 0.003 |
| Male factors | | | 443(17.3)^a^ | 15(10.6)^a^ | 681(22.0) | <0.001 |
| Combined factors | | | 32(1.2) | 0(0.0) | 52(1.7) | 0.173 |
| Others | | | 303(11.9) | 27(19.1)^ab^ | 340(11.0) | 0.010 |
| **Type of fertilization** | | |  |  |  |  |
| IVF | | | 1830(71.7)^a^ | 107(75.9)^a^ | 2022(65.2) | <0.001 |
| ICSI | | | 724(28.3) | 34(24.1) | 1080(34.8) |  |

Data are mean ±SD, median (interquartile), or n (%). ^a^p<0.05, vs. Control 1; ^b^p<0.05, vs. POSEIDON group 1.
