# Supplementary material for: Maternal and neonatal complications after IVF/ICSI-fresh embryo transfer in low-prognosis women under the POSEIDON criteria: a retrospective cohort study
Source: BMC Pregnancy Childbirth. 2023 Dec 12;23:855. doi: 10.1186/s12884-023-06176-2 (PMC10714626; doi:10.1186/s12884-023-06176-2)
Supplement: Supplementary file 4 — Additional file 4. [file 12884_2023_6176_MOESM4_ESM.docx]

**Supplement table 4** IVF/ICSI-technique parameters of participants < 35 years

|  | POSEIDON Group 1 (n=2554) | POSEIDON Group 3 (n=141) | Control 1 (n=3102) | P value |
| --- | --- | --- | --- | --- |
| **Ovarian stimulating protocol, n (%)** | |  |  | <0.001 |
| Long GnRH agonist | 1434(56.1)^a^ | 17(12.1)^ab^ | 2218(71.5) | <0.001 |
| Short GnRH agonist | 594(23.3)^a^ | 81(57.4)^ab^ | 330(10.6) | <0.001 |
| GnRH antagonist | 472(18.5)^a^ | 14(9.9)^b^ | 506(16.3) | 0.007 |
| Others | 54(2.1) | 29(20.6)^ab^ | 48(1.5) | <0.001 |
| **HCG day E_2_ (pg/mL)** | 2140(1632, 2818)^a^ | 1355(648, 1859)^ab^ | 3257(2637, 4300) | <0.001 |
| **HCG day P (ng/mL)** | 0.7(0.5, 0.9)^a^ | 0.6(0.4, 0.9)^ab^ | 0.8(0.6, 1.1) | <0.001 |
| **HCG day endometrial thickness (cm)** | 1.11±0.19^a^ | 1.09±0.23 | 1.13±0.25 | 0.009 |
| **Number of retrieved oocytes** | 7(5, 8)^a^ | 3(2, 5)^ab^ | 13(11, 15) | <0.001 |

Data are mean ±SD, median (interquartile), or n (%). ^a^p<0.05, vs. Control 1; ^b^p<0.05, vs. POSEIDON group 1.
