# Supplementary material for: Maternal and neonatal complications after IVF/ICSI-fresh embryo transfer in low-prognosis women under the POSEIDON criteria: a retrospective cohort study
Source: BMC Pregnancy Childbirth. 2023 Dec 12;23:855. doi: 10.1186/s12884-023-06176-2 (PMC10714626; doi:10.1186/s12884-023-06176-2)
Supplement: Supplementary file 5 — Additional file 5. [file 12884_2023_6176_MOESM5_ESM.docx]

**Supplement table 5** Baseline characteristics of participants ≥ 35 years

|  | POSEIDON Group 2(n=971) | POSEIDON Group 4(n=142) | Control 2 (n=718) | P value |
| --- | --- | --- | --- | --- |
| **Age (years)** | 37.4±2.3^c^ | 38.3±2.5^cd^ | 37.1±2.0 | <0.001 |
| **AFC** | 10(8, 14)^c^ | 3(2, 4)^cd^ | 13(10, 16) | <0.001 |
| **AMH (ng/mL)** | 2.3(1.7, 3.5)^c^ | 0.4(0.2, 0.8)^cd^ | 3.4(2.4, 4.9) | <0.001 |
| **FSH (IU/L)** | 7.1(6.0, 8.2)^c^ | 8.7(6.9, 11.5)^cd^ | 6.4(5.7, 7.5) | <0.001 |
| **LH (IU/L)** | 4.4(3.4, 5.7) | 4.5(3.2, 6.1） | 4.4(3.4, 5.8) | 0.663 |
| **E_2_ (pg/mL)** | 35.0(27.1, 47.8)^c^ | 42.3(26.1, 72.3)^cd^ | 32.2(24.1, 43.4) | <0.001 |
| **TO (ng/dL)** | 20.0(14.1, 26.2) | 18.3(12.5, 25.8) | 19.6(14.1, 26.8) | 0.244 |
| **TSH (μIU/mL)** | 2.1(1.4, 2.8) | 2.2(1.6, 3.0) | 2.1(1.5, 2.8) | 0.183 |
| **Basal systolic blood pressure (mmHg)** | |  |  |  |
|  | 117.2±12.0 | 115.4±13.0 | 117.7±11.7 | 0.163 |
| **Basal diatolic blood pressure (mmHg)** | |  |  |  |
|  | 70.7±9.2 | 69.3±9.1^c^ | 71.2±8.7 | 0.084 |
| **Basal blood glucose (mmol/l)** | |  |  |  |
|  | 5.26±0.51 | 5.28±0.46 | 5.30±0.44 | 0.165 |
| **BMI (kg/m^2^)** |  |  |  | 0.292 |
| BMI<18.5 | 19(2.0) | 2(1.4) | 6(0.8) | 0.162 |
| 18.5≤BMI<23 | 383(39.5) | 56(39.4) | 288(40.1) | 0.960 |
| 23≤BMI<27.5 | 431(44.4) | 56(39.4) | 309(43.0) | 0.515 |
| BMI≥27.5 | 138(14.2) | 28(19.7) | 115(16.0) | 0.192 |
| **Type of infertility** |  |  |  |  |
| Primary | 220(22.7) | 34(23.9) | 143(19.9) | 0.318 |
| Secondary | 751(77.3) | 108(76.1) | 575(80.1) |  |
| **Causes of infertility** |  |  |  | 0.121 |
| Tubal factors | 688(70.9) | 93(65.5) | 517(72.0) | 0.296 |
| Male factors | 120(12.4) | 13(9.2) | 93(13.0) | 0.454 |
| Combined factors | 14(1.4) | 4(2.8) | 11(1.5) | 0.479 |
| Others | 149(15.3) | 32(22.5)^c^ | 97(13.5) | 0.023 |
| **Type of fertilization** |  |  |  |  |
| IVF | 707(72.8) | 105(73.9) | 525(73.1) | 0.958 |
| ICSI | 264(27.2) | 37(26.1) | 193(26.9) |  |

Data are mean ± SD, median (interquartile), or n (%). ^c^p<0.05, vs. Control 2; ^d^p<0.05, vs. POSEIDON group 2.
