# Supplementary material for: Maternal and neonatal complications after IVF/ICSI-fresh embryo transfer in low-prognosis women under the POSEIDON criteria: a retrospective cohort study
Source: BMC Pregnancy Childbirth. 2023 Dec 12;23:855. doi: 10.1186/s12884-023-06176-2 (PMC10714626; doi:10.1186/s12884-023-06176-2)
Supplement: Supplementary file 6 — Additional file 6. [file 12884_2023_6176_MOESM6_ESM.docx]

**Supplement table 6** IVF / ICSI-technique parameters of participants ≥ 35 years

|  | POSEIDON Group 2 (n=971) | POSEIDON Group 4 (n=142) | Control 2 (n=718) | P value |
| --- | --- | --- | --- | --- |
| **Ovarian stimulating protocol, n (%)** | |  |  | <0.001 |
| Long GnRH agonist | 445(45.8)^c^ | 22(15.5)^cd^ | 492(68.5) | <0.001 |
| Short GnRH agonist | 406(41.8)^c^ | 69(48.6)^cd^ | 167(23.3) | <0.001 |
| GnRH antagonist | 102(10.5)^c^ | 14(9.9)^d^ | 51(7.1) | 0.053 |
| Others | 18(1.9) | 37(26.1)^c^ | 8(1.1) | <0.001 |
| **HCG day E_2_ (pg/mL)** | 2190(1680, 2864)^c^ | 1062(492, 1642)^cd^ | 3450(2690, 4409) | <0.001 |
| **HCG day P (ng/mL)** | 0.7(0.5, 0.9)^c^ | 0.6(0.3, 0.8)^cd^ | 0.8(0.6, 1.0) | <0.001 |
| **HCG day endometrial thickness (cm)** | 1.08±0.20^c^ | 1.01±0.18^cd^ | 1.12±0.20 | <0.001 |
| **Number of retrieved oocytes** | 7(5, 8)^c^ | 3(2, 5)^cd^ | 12(11, 14) | <0.001 |

Data are mean ± SD, median (interquartile), or n (%). ^c^p<0.05, vs. Control 2; ^d^p<0.05, vs. POSEIDON group 2.
